# Parent’s Perspective towards Child COVID-19 Vaccination: An Online Cross-Sectional Study in Mexico

**DOI:** 10.3390/ijerph19010290

**Published:** 2021-12-28

**Authors:** Juan Luis Delgado-Gallegos, Gerardo R. Padilla-Rivas, Lilia Julieta Gastelum-Arias, Erika Zuñiga-Violante, Gener Avilés-Rodríguez, Daniel Arellanos-Soto, Héctor Franco-Villareal, Elsa N. Garza-Treviño, María de los Ángeles Cosío-León, Gerardo Salvador Romo-Cardenas, Javier Ramos-Jiménez, Ana Ma. Rivas-Estrilla, Jorge E. Moreno-Cuevas, Jose Francisco Islas

**Affiliations:** 1 Departamento de Bioquímica y Medicina Molecular, Facultad de Medicina, Universidad Autónoma de Nuevo León, Monterrey 64460, Mexico; juan_luisdg@hotmail.com (J.L.D.-G.); gerardo.padillarv@uanl.edu.mx (G.R.P.-R.); lilii.gastelum@hotmail.com (L.J.G.-A.); d_arellanos_s@yahoo.com (D.A.-S.); egarza.nancy@gmail.com (E.N.G.-T.); javier.ramosjm@uanl.edu.mx (J.R.-J.); ana.rivasst@uanl.edu.mx (A.M.R.-E.); 2 Facultad de Ingeniería y Tecnología, Universidad de Montemorelos, Montemorelos 67515, Mexico; erikaz@um.edu.mx; 3 Facultad de Ingeniería, Arquitectura y Diseño, Universidad Autónoma de Baja California, Ensenada 22860, Mexico; gener.aviles@uabc.edu.mx (G.A.-R.); romo.gerardo@uabc.edu.mx (G.S.R.-C.); 4 Althian Clinical Research, Monterrey 64060, Mexico; dr.hectorfranco@gmail.com; 5 Dirección de Investigación, Innovación y Posgrado, Universidad Politécnica de Pachuca, Zempoala 43830, Mexico; ma.cosio.leon@upp.edu.mx; 6 Departamento de Ciencias Básicas, Universidad de Monterrey, San Pedro Garza García 66238, Mexico

**Keywords:** COVID-19 vaccine, children vaccination, México, vaccine hesitancy

## Abstract

COVID-19 vaccination programs continue in child populations. Thus, parents’ attitude towards COVID-19 vaccination of their children is crucial for these strategies to succeed. The present study derives from the application of an online COVID-19 Vaccine Acceptance & Hesitancy Questionnaire (COV-AHQ) in which we measure parent’s hesitancy towards children’s vaccination (section 4 of the COV-AHQ) and other significant factors. A logistic regression analysis with backward stepwise method was used to quantify the associations between factors and parent’s hesitancy. According to the correlation analysis, the most representative factors predicting vaccine hesitancy/acceptance were positive attitude towards vaccination, parents believing that the COVID-19 vaccine will enhance the economic situation of the country, parents actively researching information, having the willingness to obtain the COVID-19 vaccine themselves, and the possibility of their children developing adverse effects. Our findings also showed that parents are highly interested in having their children vaccinated. Nonetheless, parents expressed high levels of concern involving their children in developing adverse effects from the vaccine. In addition, obtaining influenza immunization prompted interest in obtaining the COVID-19 vaccine, and younger-aged parents are much more concerned with having their children vaccinated. Therefore, in order to ensure successful vaccination programs, policymakers and health authorities should design strategies to gain confidence and provide security amongst the population, including giving continuous information about the benefits of vaccination and presenting the frequency of side effects to bring parents on board with vaccinating their children.

## 1. Introduction

As coronavirus disease 2019 (COVID-19) continues to grow worldwide, several strategies continue to be emplaced to prevent its spread, such as quarantine, social restrictions, and use of personal protective equipment [1,2,3]. While uncertainty continues as to the potential extent of the disease, a crucial step is to help enhance the immune system, hence the need for the development of vaccines to suit such purpose [4,5,6]. 

COVID-19´s high infection rates around the world have added pressure to the need to develop vaccines, particularly as new variants of the virus emerge, as well as to enhance distribution and access to them. Given today’s technological advances, leaps are being made to advance and manufacture novel models of vaccine at an unprecedented pace [7,8]. Therefore, we come to the need to enhance vaccine awareness as a critical step in addressing the barriers associated with its comprehension, benefits, and possible adverse effects [9]. The latter is of particular concern, especially when dealing with groups of interest such as children, as research is still in its early stages to understand potential complications and mid- to long-term effects [10,11,12]. 

In April 2020, the World Health Organization issued a statement: “Immunization saves millions of lives every year and it is widely recognized as one of the world’s most successful and cost-effective health interventions”. Vaccines and vaccination programs have proven their beneficial effects and efficiency by reducing morbidity and mortality of several preventable diseases and preventing around 2–3 million deaths every year [13]. Vaccines, in particular novel ones for COVID-19, have permitted the earliest return of the workforce to the labor market and have given an added layer of protection to the healthcare sector which continues to work diligently treating patients, regardless of the physical, emotional, and psychological impact COVID-19 has had on them [8,13,14,15]. Therefore, vaccines not only aid in the economy’s boost but also reduce healthcare costs, promote healthcare equity, and increase the potential caregiver’s productivity [9,16,17]. Despite the benefits vaccination brings, there is still a possibility that after becoming immunized one could develop adverse side effects, such as self-limited local pain, fever, dizziness, tachycardia, muscle ache, and the more uncommon potential to develop Guillain-Barré syndrome and other life-threatening allergic reactions. Recently, the CDC reported that 16:1,000,000 people developed Guillain-Barre syndrome and 2 to 5:1,000,000 people developed anaphylaxis, secondary to vaccination. The risk of presenting any of these symptoms, especially the more uncommon or dangerous such as Guillain-Barré syndrome, may lead to an increase in fear of vaccines in certain parts of the general population [11,18]. Unfortunately, several groups have used some of these conditions to justify not becoming vaccinated. In addition, some individuals have also popularized the idea that children pose no real threat to the public if the rest of the population vaccinates. Ironically, this argument requires that most of the public are vaccinated. Overall, the refusal of vaccination opens channels for viral spread, enhancing the pressure of spread/infection for other individuals. This becomes an important issue, particularly when dealing with children whom, if infected, can easily become vectors for viral spreading [12,19,20]. 

Vaccine hesitancy has been defined as “a complex and context-specific delay in acceptance or refusal of vaccination despite availability influenced by factors such as complacency, convenience, and confidence”, resulting in delay or refusal of vaccination [7,21,22]. From the parental perspective, hesitancy in vaccinating their children can arise from several key factors, such as their socioeconomic status, education, healthcare access, and attitudes related to vaccination [12,21]. Beliefs such as children’s immune systems being overloaded by the amount of vaccines they receive, thinking the vaccine is more dangerous than the illness, religious beliefs, distrust of public healthcare systems, government manipulation, adverse side effects, costs of vaccination, knowing someone else experiencing a side effect, or misunderstanding of the vaccine components have all been associated with vaccine refusal [23]. As previously mentioned, hesitancy towards vaccination has become a common phenomenon, prompting the WHO Strategic Advisory Group of Experts on Immunization or SAGE to categorize “vaccine hesitancy” as one of the top 10 public health treats [22].

According to recent studies evaluating parents’ attitude towards influenza vaccination in the current pandemic, the fear of COVID-19 in minors can be a prime motivation for vaccinating children. In the studied populations, results showed that the attitude towards vaccination is comparable to that from influenza H1N1 [10,11]. Therefore, annual vaccination patterns could be comparable to those of COVID-19, given the vaccines availability. 

Although a global matter, almost all of the studies evaluating parents’ attitudes towards vaccination have been carried out in the United States, Canada, and United Kingdom [10,24], therefore there is an important need to analyze Latin America. In Mexico, as of 21 November 2021, according to official government COVID-19 records, COVID-19 had afflicted over 3,862,137 confirmed cases, from which there are 289,419 (7.5%) of total cases reported for ages 0 to 19 [25,26]. Given the socioeconomic and cultural similarities of Mexico and the rest of Latin America, we opted to use a Mexican sample of parents as an initial model to understand vaccine acceptance and hesitancy, based on a general COVID-19 Vaccine Acceptance & Hesitancy Questionnaire (COV-AHQ) which included many of the factors earlier mentioned, such as religion, socioeconomic status, education, personal beliefs about vaccines, and other importer factors which can contribute to parental hesitancy [12,21,23]. 

## 2. Materials and Methods

### 2.1. Questionnaire COV-AHQ 

The present study derives from the application of the online COVID-19 Vaccine Acceptance & Hesitancy Questionnaire, or COV-AHQ (Cronbach’s alpha > 0.8) [27], which was designed based on the initial and Adapted COVID-19 Stress Scales (CSS and ACSS) [28,29,30] and the Vaccine Hesitancy Scales (VHS) [22]. For the present study, we took into consideration only the subpopulation of participants with the criteria of answering yes to having children. 

The COV-AHQ was written using MS Forms (Microsoft Corporation, Redwood, WA, United States) which conveniently permitted distribution and application via the following web-link https://forms.office.com/r/QhkS5wjzMM We divided the questionnaire into six important sections; an initial sociodemographic section, followed by sections 1 through 4 analyzing four psychometric areas: section 1 (danger and contamination), section 2 (xenophobia), section 3 (fear of vaccination’s adverse effects), section 4 (parent’s hesitancy towards children’s vaccination), and finally, a general COVID-19 questions section. For sections 1 to 4, a Likert-scale format with increasing point value was used to classify answers. Using this system, we classified the resulting scores to the following scales: absent 0 to 4, mild 5 to 8, moderate 9 to 12, severe 13 to 16.

### 2.2. Participants 

To calculate a representative sample size for parents in Mexico, we took into consideration that the probability that parents would have opposing views would be 50% (*p* value of 0.5), considering that the variable behavior is binomial. Using the classical formula for sample sizes, an interval of confidence of 99% (z value of 2.58) and a margin of error of 5% (delta value of 0.05) was used, which resulted in *n* = 666 participants [31]. 

### 2.3. Answers and Frequencies

We calculated the frequency of answers for each question in the sections and for other sociodemographic variables such as occupation, gender, age, practice of religion, education level, total of habitants in household, total number of rooms in household, comorbidity, knowing someone positive for COVID-19, attitude towards vaccination, previous influenza vaccination for the 2020–2021 season, consideration of a COVID-19 vaccine as a positive to the current socioeconomic situation of the country, willingness to receive the COVID-19 vaccination, and participants actively researching COVID-19 vaccine information.

### 2.4. Data Analysis

We then followed with a logistic regression analysis using statements of section 4 (directly applicable to child vaccination).

1. I consider that getting my child vaccinated is important for the health of others in my community.
2. I consider that getting my child vaccinated is a good protective measure.
3. I am concerned about my child developing an adverse effect related to the COVID vaccination.

Regression analysis took into consideration sociodemographic variables such as age group, gender, having comorbidities, level of education, and having received the influenza vaccine for the 2020–2021 season. The analysis introduced these selected factors and the sections into a backward stepwise (likelihood ratio) method. Finally, we used the results to quantify the associations between factors and sections to the statements of section 4, obtaining unstandardized regression coefficients (B), odds ratios (ORs), and their 95% confidence intervals (CIs). We further calculated Pearson’s chi-squared and an R ratio of 0.05, all statistical analysis using IBM-SPSS Statistics for Windows, Version 23.0 (IBM Corp., Armonk, NY, USA). 

### 2.5. Questionnaire Distribution

The COV-AHQ was distributed to the general population in Mexico through emails and social media such as Twitter, Reddit, and Facebook, from the period of December 2020 to February 2021; the period before the beginning of the official vaccination program in Mexico [25]. To participate, all subjects had to acknowledge being >18 years of age and give consent to participate via the electronic form. 

### 2.6. Institutional Review Board Statement

We conducted the study under the Declaration of Helsinki, and the Ethics Committee of Hospital La Misión, Monterrey NL. México approved the protocol. Protocol # VAC-CAMCVC-01. 

## 3. Results

The results of the COV-AHQ showed 699 participants self-responded as having children. From the cohort of parent participants, in Table 1 we present their sociodemographic profile. Briefly, the data showed respondents were 69.1% females and 27% males. Most participants (66.8%) were in the age group of 34 to 54 years. Interestingly, over 93% of participants reported as being actively working, while 5.3% reported being currently unemployed; albeit participants were not asked if they were looking or were not interested in working. An almost 1:2 ratio of participants answered having a comorbidity (36.2%). Most participants reported as professionally educated, with 51.2% reported as having at least a bachelor’s degree and an additional 35.8% as having graduate studies. House occupancy mostly centered on families of four individuals (34.6%). Finally, most individuals (69.5%) answered as having over four rooms in their house (excluding kitchen and bathroom).

In another section of the survey, participants were asked to answer general questions about COVID-19. One striking fact was that 97.4% answered “yes” to knowing someone with a previous COVID-19 diagnosis; meanwhile, to having had, themselves, a previous COVID-19 positive diagnosis, 84% answered “no”. Over 78% of participants had a positive attitude towards vaccination. This seems to fall in line with the 87.4% of participants answering positively to their willingness to become vaccinated. Another interesting fact was that 87.1% of parents agreed that obtaining the vaccine will enhance the overall economic situation of the country. Additionally, it was asked whether participants had received an influenza vaccine for the 2020–2021 flu season. Here, results showed that 43.5% of participants had received it. 

**Figure 1:**
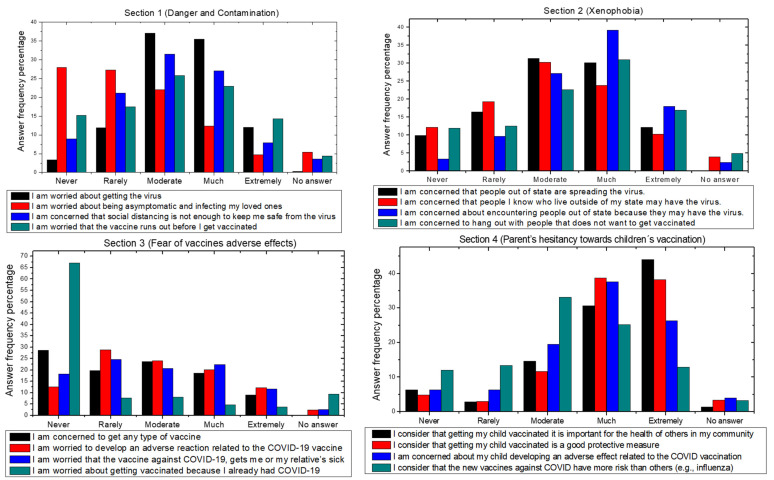
Individual answers by question per section.

Next, participants answered the four sections of the COV/AHQ, and from the results (Table 2) we can observe for section 1 that 41.1% (*n* = 287) fell in the “mild” classification, as the most prevalent classification. For section 2, most participants 39.5% (*n* = 276) fell in the moderate classification, while for section 3, the majority 43.3% (*n* = 303) fell in the “absent” classification; finally, in section 4, the grand majority 56.1% (*n* = 392) fell under “moderate”. Participants’ responses to individual questions are shown in Table 3 and Figure 1.

Using the sociodemographic variables such as age group, gender, having comorbidities, level of education, having received the influenza vaccine for the 2020–2021 season, and the individual statements directly related to children’s vaccination of section 4 (1. I consider that getting my child vaccinated is important for the health of others in my community, 2. I consider that getting my child vaccinated is a good protective measure, 3. I am concerned about my child developing an adverse effect related to the COVID vaccination), as they relate to parents’ hesitancy towards children’s vaccination, we performed, using SPSS ^®^ software, a binary logistic regression analysis, as seen in Table 4. By running the algorithm, we could pluck, in a stepwise manner, the variables that did not influence the outcome. 

Results for “I consider that getting my child vaccinated is important for the health of others in my community” showed that section 2 did not have any effect; meanwhile, the following variables all seem to have an influence: section 1 “absent” (*p* < 0.001, OR:0.189) and “mild” (*p* < 0.007, OR:0.448), section 3 “absent” (*p* < 0.001, OR:24.71), “mild” (*p* < 0.007, OR:5.504), and “moderate” (*p* < 0.044, OR:3.539), and section 4 “absent” (*p* < 0.001, OR:0.007), “mild” (*p* < 0.001, OR:0.014), and “moderate” (*p* < 0.001, OR:0.1), and having had the influenza vaccination during the 2020–2021 season (*p* < 0.001, OR:2.173), all with a Nagelkerke R-Squared of 0.445. Individual step analysis and cross table are shown in Supplemental Table S1.

For “I consider that getting my child vaccinated is a good protective measure”, all four sections and the following variables seem to have an influence: section 1 “absent” (*p* < 0.001, OR:0.252) and “mild” (*p* < 0.022: OR:0.447), section 2 “absent” (*p* < 0.001, OR:0.305), section 3 “absent” (*p* < 0.001, OR:14.195) and “mild” (*p* < 0.004, OR:2.621), section 4 “absent” (*p* < 0.001, OR:0.001), “mild” (*p* < 0.001, OR:0.008), and moderate (*p* < 0.001, OR:0.056), age groups 18 to 34 years (*p* < 0.04, OR:0.35) and 35 to 54 (*p* < 0.007, OR:0.32), and having had the influenza vaccination during the 2020–2021 season (*p* < 0.001, OR:2.744), all with a Nagelkerke of 0.528. Individual step analysis and cross table are shown in Supplemental Table S2.

For “I am concerned about my child developing an adverse effect related to the COVID vaccination”, section 1 did not seem to have an effect, while the following variables all seem to have an influence: section 2 “absent” (*p* < 0.004, OR:3.12), “mild” (*p* < 0.001, OR:3.936), and “moderate” (*p* < 0.016, OR:2.223), section 3 “absent” (*p* < 0.001, OR:0.051) and “mild” (*p* < 0.001, OR:0.267), section 4 “absent” (*p* < 0.001, OR:0.011), “mild” (*p* < 0.001, OR:0.03), and “moderate” (*p* < 0.001, OR:106), and age group 18 to 34 years (*p* < 0.01, OR:0.47), all with a Nagelkerke of 0.462. Individual step analysis and cross table are shown in Supplemental Table S3.

The regression model brought attention to the fact that participants categorized as severe, those we thought beforehand would have a great weight in the model, were eliminated after step 1. We further calculated Pearson´s correlation between all four sections (categories), as seen in Supplemental Table S4. Section 1 (severe) seems to have the highest correlation to section 1 (mild) with r = −0.270 and *p* < 0.001, section 2 (severe) correlates the highest with section 2 (moderate) with r = −0.366 and *p* < 0.001, section 3 (severe) correlates the highest with section 3 (absent) r = −0.195 and *p* < 0.001, and finally section 4 (severe) correlates the highest with section 4 (moderate) r = −0.651 and *p* < 0.001.

## 4. Discussion

Mexico continues with its vaccination programs and public health strategies to prevent the spread of COVID-19 [32], and it is in the early stages of the COVID-19 children’s vaccination programs [33]. As of 21 November 2021, approximately 58% of the total population in Mexico has had at least one vaccine dose [34]. Although Mexico has had a long history of successful massive vaccination programs, distrust and uncertainty in the current vaccines have upsurged [7,11]. This uncertainty could play an important role when parents face the dilemma of having to immunize their children, hence the importance of studying the factors that can drive vaccine hesitancy, particularly in parents.

From a parental perspective, 78.5% of parents seem to have a positive attitude towards vaccination, with 87.1% of parents believing that the COVID-19 vaccine will enhance the economic situation of the country, 73.5% of parents actively researching information on the subject, and 87.4% having the willingness to obtain the COVID-19 vaccine themselves. Yet, only close to half of the surveyed parents (43.5%) had actually been vaccinated for influenza; a potential model for COVID-19 vaccination intention [35,36]. We should state that influenza immunizations during the 2020 season were reported to have tripled in Mexico, under the potential perspective that the vaccine could help reduce COVID-19 symptoms [37]. We could relate work as one potential reason for the results showing less than half of the surveyed population becoming immunized, particularly those with busy schedules, as only 6.7% of individuals reported currently not working as either unemployed or as active students. Another important factor to consider for the lack of vaccinated parents was that there was a limited availability of the influenza vaccine at the beginning of the vaccination season. Mexico had a guidance to prioritize influenza vaccine for children and older adults, leaving 18–64 years old adults without prompt access to the influenza vaccine. Comparably, the recent report for the 2020 influenza season showed that in the US, 52.2% of adults (40% average for the Hispanic population) had been vaccinated [38]. Remarkably, when we take a more in-depth look at parental responses from the survey, 72.6% of responders had “moderate to much” worry of themselves contracting the disease, with 12% being extremely worried, while 58.5% of responders were “moderate to much” concerned that social distancing was not enough to keep one safe, and 48.6% were “moderate to much”, with 14.3% being extremely, worried that they would not obtain the vaccine because of fear it would run out. This, theoretically, separates the notion that influenza vaccination is a good predictor for COVID-19 vaccine acceptance. It is important to note that these questions were directed at the parents, who are the caregivers, hence having direct emotional, social, and economical ties to their children. Could parents be more concerned about being able to support their families, rather than potential effects of the vaccine? Or could they have the thought “if something happens to me, how is my family going to be supported?” Conversely to our expectations, parents responded mostly 52.8% “rarely to moderate” to their worry about developing adverse effects from the vaccines. 

Another interesting remark amongst parents was their responses to questions related to xenophobia, as 56.5% of individuals scored “moderate to severe”, meaning there is a notable fear of people from outside the local community to come in and potentially spread the virus. When asked about their concern of people outside their local state bringing in the virus, 54.5% answered “much to moderate”, with an extra 10.3% extremely worried. An additional concern was seen when asked about encountering people outside their local state, with 66.4% of respondents answering “much to moderate,” and an extra 18% being extremely worried. These results mirror the global sentiment which is worrisome given the present harassment towards individuals of Asian descent, as they have been wrongly labeled as primal carriers with severe violent racially motivated violence against them [39,40]. Similar xenophobic behaviors have been observed all over the globe, yet they seem to be reflected mostly in the most liberal economies: Australia, France, Germany, and UK, where antimigration groups founded on social and economic basis feel threatened by the outside labor force [38]. Unfortunately, during the highest peaks of the pandemic, many businesses were affected, and others had to shut down, bringing a sense of unease to the working professional, and as a result many families have been economically affected, potentially adding an extra burden [41,42]. Given this global panorama, and the burdens left by COVID-19, it is understandable that parents can be highly concerned.

In our study, section 4 dedicated to the parents´ hesitancy towards children’s vaccination was studied using a logistic regression model looking at how the social demographic variables, along with the sections themselves, would prove relevant. The model considers all variables and begins a stepwise removal of all nonsignificant and redundant variables. As we have stated, section 4 had three questions directly pertaining to children’s vaccination, which were used independently as anchors to run the regression. From the first statement “I consider that getting my child vaccinated is important for the health of others in my community”, there seems to be no contribution from section 2 (xenophobia), as the question relates to improving the community. We see this result as fascinating, as we had earlier mentioned that parents seem to be highly concerned about people coming with the disease and spreading it; hence, having a layer of protection for their children such as the vaccine would be most desirable. One interesting variable that emerged was having had an influenza immunization, as it is important not only to have the willingness to become vaccinated, but to actually go through it in order to help protect others in the community. For the second statement, “I consider that getting my child vaccinated is a good protective measure”, all sections seem to be relevant, as well as the effect of becoming vaccinated for influenza. Interestingly, we also see that both young (18 to 34 years) and mid-age (35 to 54 years) parents present relevancy. Although older active adults are most likely concerned about child infections, many studies have shown that the initial most-at-risk group was actually the older adults, particularly those with comorbidities [30,38,43,44,45,46]. Therefore, it is most likely that older active adults are currently more worried about their own health. Finally, for the statement “I am concerned about my child developing an adverse effect related to the COVID vaccination”, here we see that section 1 (danger and contamination) does not have relevancy, as this section is more about contracting the virus, spreading it, and being asymptomatic; it again becomes understandable that no direct relation is warranted. Young parents (18 to 34 years) have more concerns over the potential adverse effects—a potential fear that parents have [47], and while this fear is not to be dismissed, it is important that more longitudinal studies continue to provide more valuable information on potential adverse effects, as well as long-term ones. We should remember that children’s immune systems are not those of young adults, they are even more vigorous [47,48], and, therefore, we should expect more reactivity to similar amounts of vaccine, prompting more fine-tuning in future studies.

## 5. Limitations

Because of the COVID-19 quarantine and social distancing restrictions, we applied the COV-AHQ using a digital platform (Microsoft Forms). We could consider this as a limitation as it is not supervised in person. Remote evaluations provide a safe alternative, albeit relying on the inclination to answer.

Currently, online questionnaires have been a valuable tool used by several researchers, particularly in the COVID-19 pandemic, to gather public or selected group information [14,22,35,49,50,51,52]. We distributed the questionnaire either by direct email invitation or by popular social media platforms, and we further asked participants to send other colleagues or acquaintances the link to the questionnaire. However, this poses the problem of not being able to calculate a participation rate because of the nature of the distribution in a snowball-like method. In addition, full completion of the questionnaire of participants was optional, as we believe that this might deter some from continuing. Our group distributes well among age groups, gender, types of employment, and other social demographic aspects.

## 6. Conclusions

At over a year after the outbreak of the COVID-19 pandemic, and with the current technology of vaccines at hand, we face a new dilemma: the parental perspective on having their children vaccinated. As studies have only just begun, there is still much information to be obtained and processed; however, the eminent threat of COVID-19 remains, therefore, both clinical studies and parental input are critical in moving in the right direction.

In Mexico, as of early October, COVID-19 children’s vaccination programs were set in motion. Our current findings showed that parents are highly interested in having their children vaccinated, as this is a good measure for protection for both them and their community. Parents also express high levels of concern involving their children in developing adverse effects from the vaccine. When the parental hesitancy was broken down into questions and analyzed by regression, we observed the section related to adverse effects was always present as a key variable. Prominent also was the fact that obtaining influenza immunization prompted interest in obtaining the COVID-19 vaccine, and that younger-aged parents are much more concerned with having their children vaccinated.

The lack of vaccination among children can lead them to contract preventable diseases and contribute to viral spread. Thus, the parent’s attitude towards general vaccination and COVID-19 vaccination is crucial. Therefore, to improve successful programs, strategies should be designed to gain confidence and provide security among the population; giving continuous information about the benefits of vaccination, as well as presenting the frequency of side effects to bring parents on board with vaccinating their children.

## Figures and Tables

**Table 1:** Sociodemographic profile.

Employment	*n*	%	Gender	*n*	%	Age	*n*	%
Unemployed	37	5.3	Male	188	26.9	18 to 34	110	15.7
Student	10	1.4	Female	483	69.1	35 to 54	467	66.8
Health professional	73	10.4	No answer	28	4.0	55+	122	17.5
Essential Worker	132	18.9	Total	699	100	Total	699	100
Nonessential worker	83	11.9						
Commerce	48	6.9	Practice of Religion	*n*	%	Comorbidities	*n*	%
Academic professional	136	19.5	No	136	19.5	No	446	63.8
Other	180	25.8	Yes	546	78.1	Yes	253	36.2
Total	699	100	No answer	17	2.4	No answer	0	0
			Total	699	100	Total	699	100
Education level	*n*	%	HouseholdOccupants	*n*	%	Rooms	*n*	%
Elementary	3	0.4	1	26	3.7	1	5	0.7
Jr High school	13	1.9	2	77	11.0	2	26	3.7
High school	63	9.0	3	180	25.8	3	60	8.6
Bachelors	358	51.2	4	242	34.6	4	113	16.2
Graduate	250	35.8	+4	168	24.0	+4	486	69.5
No answer	12	1.7	No answer	6	0.9	No answer	9	1.3
Total	699	100	Total	699	100	Total	699	100
Knowing someone with a previous COVID-19 diagnosis	*n*	%	Attitude towards vaccination	*n*	%	Influenza vaccine during the period of 2020–2021	*n*	%
No	11	1.6	Disagree	30	4.3	Vaccinated	304	43.5
Yes	681	97.4	Neutral	113	16.2	No vaccinated	389	55.7
No answer	7	1.0	Agree	549	78.5	No answer	6	0.9
Total	699		No answer	7	1.0	Total	699	100
			Total	699	100			
Willingness to become COVID-19 vaccinated	*n*	%	Participants actively researching COVID-19 vaccine information	*n*	%	Having had a previous COVID-19 diagnosis	*n*	%
No	79	11.3	No	175	25.04	No	587	83.98
Yes	611	87.4	Yes	514	73.53	Yes	105	15.02
No Answer	9	1.3	No answer	10	1.43	No answer	7	1.00
Total	699	100	Total	699	100	Total	699	100
The COVID-19 vaccine will enhance the economic situation	*n*	%						
Disagree	80	11.4						
Agree	609	87.1						
No answer	10	1.4						
Total	699	100						

**Table 2:** Prevalence in the individual sections (classifications).

	Section 1 Danger and Contamination		Section 2 Xenophobia		Section 3 Fear of Vaccinations Adverse Effects		Section 4 Parents’ Hesitancy towards Children’s Vaccination	
	*n*	%	*n*	%	*n*	%	*n*	%
Absent	148	21.2	104	14.9	303	43.3	29	4.1
Mild	287	41.1	200	28.6	211	30.3	104	14.9
Moderate	198	28.3	276	39.5	152	21.7	392	56.1
Severe	66	9.4	119	17	33	4.7	174	24.9
	699	100	699	100	699	100	699	100

**Table 3:** Individual question results sections.

Section 1—Danger and Contamination			Section 2—Xenophobia		
I Am Worried about Getting the Virus	*n*	%	I Am Concerned That People out of State are Spreading the Virus	*n*	%
Never	23	3.3	Never	69	9.9
Rarely	83	11.9	Rarely	115	16.5
Moderate	259	37.1	Moderate	219	31.3
Much	248	35.5	Much	211	30.2
Extremely	84	12.0	Extremely	85	12.2
No answer	2	0.3	No answer	0	0.0
Total	699	100	Total	699	100
I am worried about being asymptomatic and infecting my loved ones.	*n*	%	I am concerned that people I know who live outside of my state may have the virus.	*n*	%
Never	196	28.0	Never	85	12.2
Rarely	191	27.3	Rarely	135	19.3
Moderate	154	22.0	Moderate	212	30.3
Much	87	12.4	Much	167	23.9
Extremely	33	4.7	Extremely	72	10.3
No answer	38	5.4	No answer	28	4.0
Total	699	100	Total	699	100
I am concerned that social distancing is not enough to keep me safe from the virus	*n*	%	I am concerned about encountering people out of state because they may have the virus.	*n*	%
Never	62	8.9	Never	24	3.4
Rarely	148	21.2	Rarely	68	9.7
Moderate	220	31.5	Moderate	190	27.2
Much	189	27.0	Much	274	39.2
Extremely	55	7.9	Extremely	126	18.0
No answer	25	3.6	No answer	17	2.4
Total	699	100	Total	699	100
I am worried that the vaccine runs out before I get vaccinated	*n*	%	I am concerned to hang out with people that does not want to get vaccinated	*n*	%
Never	106	15.2	Never	83	11.9
Rarely	122	17.5	Rarely	88	12.6
Moderate	180	25.8	Moderate	158	22.6
Much	160	22.9	Much	217	31.0
Extremely	100	14.3	Extremely	119	17.0
No answer	31	4.4	No answer	34	4.9
Total	699	100	Total	699	100
Section 3—Fear of Vaccinations adverse effects			Section 4—Parent’s hesitancy towards children´s vaccination		
I am concerned to get any type of vaccine	*n*	%	I consider that getting my child vaccinated is important for the health of others in my community	*n*	%
Never	200	28.6	Never	45	6.4
Rarely	138	19.7	Rarely	20	2.9
Moderate	166	23.7	Moderate	103	14.7
Much	131	18.7	Much	214	30.6
Extremely	63	9.0	Extremely	308	44.1
No answer	1	0.1	No answer	9	1.3
Total	699	100	Total	699	100
I am worried to develop an adverse reaction related to the COVID-19 vaccine	*n*	%	I consider that getting my child vaccinated is a good protective measure	*n*	%
Never	88	12.6	Never	34	4.9
Rarely	201	28.8	Rarely	21	3.0
Moderate	168	24.0	Moderate	81	11.6
Much	141	20.2	Much	271	38.8
Extremely	85	12.2	Extremely	268	38.3
No answer	16	2.3	No answer	24	3.4
Total	699	100	Total	699	100
I am worried that the vaccine against COVID-19 makes me or my relatives sick	*n*	%	I am concerned about my child developing an adverse effect related to the COVID vaccination	*n*	%
Never	127	18.2	Never	44	6.3
Rarely	172	24.6	Rarely	44	6.3
Moderate	144	20.6	Moderate	136	19.5
Much	156	22.3	Much	263	37.6
Extremely	82	11.7	Extremely	184	26.3
No answer	18	2.6	No answer	28	4.0
Total	699	100	Total	699	100
I am worried about getting vaccinated because I already had COVID-19	*n*	%	I consider that the new vaccines against COVID have more risk than others (e.g., influenza)	*n*	%
Never	468	67.0	Never	84	12.0
Rarely	53	7.6	Rarely	94	13.4
Moderate	56	8.0	Moderate	232	33.2
Much	32	4.6	Much	176	25.2
Extremely	25	3.6	Extremely	90	12.9
No answer	65	9.3	No answer	23	3.3
Total	699		Total	699	100

**Table 4:** Stepwise regression model.

Questions		b	Std Error	Wald	gl	sig. (*p* Value)	Odds Ratio	95% C.I. for EXP(B)	
	Variables							Inferior	Superior
I consider that getting my child vaccinated is important for the health of others in my community	Section 1 (Absent)	−1.666	0.34	23.998	1	<0.001	0.189	0.097	0.368
	Section 1 (Mild)	−0.803	0.297	7.304	1	0.007	0.448	0.25	0.802
	Section 3 (Absent)	3.207	0.648	24.504	1	<0.001	24.71	6.94	87.974
	Section 3 (Mild)	1.706	0.628	7.373	1	0.007	5.504	1.607	18.852
	Section 3 (Moderate)	1.264	0.628	4.045	1	0.044	3.539	1.033	12.125
	Section 4 (Absent)	−4.928	0.725	46.216	1	<0.001	0.007	0.002	0.03
	Section 4 (Mild)	−4.293	0.507	71.684	1	<0.001	0.014	0.005	0.037
	Section 4 (Moderate)	−2.306	0.448	26.501	1	<0.001	0.1	0.041	0.24
	Influenza vaccine during the period of 2020–2021	0.776	0.232	11.202	1	<0.001	2.173	1.379	3.423
I consider that getting my child vaccinated is a good protective measure	Section 1 (Absent)	−1.379	0.415	11.045	1	<0.001	0.252	0.112	0.568
	Section 1 (Mild)	−0.805	0.351	5.242	1	0.022	0.447	0.225	0.891
	Section 2 (Absent)	−1.188	0.356	11.131	1	<0.001	0.305	0.152	0.613
	Section 3 (Absent)	2.653	0.387	46.996	1	<0.001	14.195	6.649	30.306
	Section 3 (Mild)	0.964	0.337	8.198	1	0.004	2.621	1.355	5.069
	Section 4 (Absent)	−8.155	1.308	38.887	1	<0.001	<0.001	<0.001	0.004
	Section 4 (Mild)	−4.89	0.685	50.972	1	<0.001	0.008	0.002	0.029
	Section 4 (Moderate)	−2.879	0.626	21.183	1	<0.001	0.056	0.016	0.191
	18 to 34 y	−1.049	0.51	4.226	1	0.04	0.35	0.129	0.952
	35 to 54 y	−1.139	0.425	7.186	1	0.007	0.32	0.139	0.736
	Influenza vaccine during the period of 2020–2021	1.009	0.279	13.06	1	<0.001	2.744	1.587	4.744
I am concerned about my child developing an adverse effect related to the COVID vaccination	Section 2 (Absent)	1.138	0.397	8.224	1	0.004	3.12	1.434	6.789
	Section 2 (Mild)	1.37	0.349	15.387	1	<0.001	3.936	1.985	7.806
	Section 2 (Moderate)	0.799	0.333	5.768	1	0.016	2.223	1.158	4.268
	Section 3 (Absent)	−2.978	0.359	68.779	1	<0.001	0.051	0.025	0.103
	Section 3 (Mild)	−1.32	0.375	12.36	1	<0.001	0.267	0.128	0.558
	Section 4 (Absent)	−4.529	1.009	20.136	1	<0.001	0.011	0.001	0.078
	Section 4 (Mild)	−3.496	0.467	56.116	1	<0.001	0.03	0.012	0.076
	Section 4 (Moderate)	−2.242	0.402	31.136	1	<0.001	0.106	0.048	0.233
	18 to 34 y	−0.755	0.291	6.718	1	0.01	0.47	0.266	0.832

I consider that getting my child vaccinated is important for the health of others in my community, Nagelkerke R-Squared 0.445; I consider that getting my child vaccinated is a good protective measure, Nagelkerke R-squared 0.528; I am concerned about my child developing an adverse effect related to the COVID vaccination, Nagelkerke R-Squared 0.462.

## Data Availability

The original contributions presented in the study are included in the article/Supplementary Material, further inquiries can be directed to the corresponding author.

## Supplementary Materials

The following supporting information can be downloaded at: https://www.mdpi.com/article/10.3390/ijerph19010290/s1. Supplemental Table S1. Logistic regression analysis with backward stepwise I consider that getting my child vaccinated is important for the health of others in my community (reglog14). Supplemental Table S2. Logistic regression analysis with backward stepwise for I consider that getting my child vaccinated is a good protective measure (reglog16). Supplemental Table S3. Logistic regression analysis with backward stepwise for I am concerned about my child developing an adverse effect related to the COVID vaccination (reglog18). Supplemental Table S4. Correlation between sections.

## References

[1] Liu M., Cheng S.-Z., Xu K.-W., Yang Y., Zhu Q.-T., Zhang H., Yang D.-Y., Cheng S.-Y., Xiao H., Wang J.-W. (2020). Use of personal protective equipment against coronavirus disease 2019 by healthcare professionals in Wuhan, China: Cross sectional study. BMJ.

[2] Park C.L., Russell B.S., Fendrich M., Finkelstein-Fox L., Hutchison M., Becker J. (2020). Americans’ COVID-19 Stress, Coping, and Adherence to CDC Guidelines. J. Gen. Intern. Med..

[3] Shah K., Kamrai D., Mekala H., Mann B., Desai K., Patel R.S. (2020). Focus on Mental Health During the Coronavirus (COVID-19) Pandemic: Applying Learnings from the Past Outbreaks. Cureus.

[4] HU (2021). Mortality Analyses. https://coronavirus.jhu.edu/data/mortality.

[5] Sohrabi C., Alsafi Z., O’Neill N., Khan M., Kerwan A., Al-Jabir A., Iosifidis C., Agha R. (2020). World Health Organization declares global emergency: A review of the 2019 novel coronavirus (COVID-19). Int. J. Surg..

[6] Albott C.S., Wozniak J.R., McGlinch B.P., Wall M.H., Gold B.S., Vinogradov S. (2020). Battle Buddies: Rapid Deployment of a Psychological Resilience Intervention for Health Care Workers During the COVID-19 Pandemic. Anesth. Analg..

[7] Feleszko W., Lewulis P., Czarnecki A., Waszkiewicz P. (2021). Flattening the Curve of COVID-19 Vaccine Rejection—An International Overview. Vaccines.

[8] Shah K., Chaudhari G., Kamrai D., Lail A., Patel R.S. (2020). How Essential Is to Focus on Physician’s Health and Burnout in Coronavirus (COVID-19) Pandemic?. Cureus.

[9] Trivedi D. (2014). Cochrane Review Summary: Face-to-face interventions for informing or educating parents about early childhood vaccination. Prim. Health Care Res. Dev..

[10] Goldman R.D., Yan T.D., Seiler M., Cotanda C.P., Brown J.C., Klein E.J., Hoeffe J., Gelernter R., Hall J.E., Davis A.L. (2020). Caregiver willingness to vaccinate their children against COVID-19: Cross sectional survey. Vaccine.

[11] Spencer J.P., Pawlowski R.H.T., Thomas S. (2017). Vaccine Adverse Events: Separating Myth from Reality. Am. Fam. Physician.

[12] Rosso A., Massimi A., De Vito C., Adamo G., Baccolini V., Marzuillo C., Vacchio M.R., Villari P. (2019). Knowledge and attitudes on pediatric vaccinations and intention to vaccinate in a sample of pregnant women from the City of Rome. Vaccine.

[13] World Health Organization Immunization Coverage. https://www.who.int/news-room/fact-sheets/detail/immunization-coverage.

[14] Barzilay R., Moore T.M., Greenberg D.M., DiDomenico G.E., Brown L.A., White L.K., Gur R.C., Gur R.E. (2020). Resilience, COVID-19-related stress, anxiety and depression during the pandemic in a large population enriched for healthcare providers. Transl. Psychiatry.

[15] Blake H., Bermingham F., Johnson G., Tabner A. (2020). Mitigating the Psychological Impact of COVID-19 on Healthcare Workers: A Digital Learning Package. Int. J. Environ. Res. Public Health.

[16] Soofi M., Najafi F., Karami-Matin B. (2020). Using Insights from Behavioral Economics to Mitigate the Spread of COVID-19. Appl. Health Econ. Health Policy.

[17] Detoc M., Bruel S., Frappe P., Tardy B., Botelho-Nevers E., Gagneux-Brunon A. (2020). Intention to participate in a COVID-19 vaccine clinical trial and to get vaccinated against COVID-19 in France during the pandemic. Vaccine.

[18] CDC Se Notificaron Algunas Reacciones Adversas Después de la Vacunación Contra el COVID-19. https://espanol.cdc.gov/coronavirus/2019-ncov/vaccines/safety/adverse-events.html.

[19] Dyda A., King C., Dey A., Leask J., Dunn A.G. (2020). A systematic review of studies that measure parental vaccine attitudes and beliefs in childhood vaccination. BMC Public Health.

[20] Hardt K., Bonanni P., King S., Santos J.I., El-Hodhod M., Zimet G.D., Preiss S. (2016). Vaccine strategies: Optimising outcomes. Vaccine.

[21] Olson O., Berry C., Kumar N. (2020). Addressing Parental Vaccine Hesitancy towards Childhood Vaccines in the United States: A Systematic Literature Review of Communication Interventions and Strategies. Vaccines.

[22] Shapiro G.K., Tatar O., Dubé E., Amsel R., Knauper B., Naz A., Perez S., Rosberger Z. (2018). The vaccine hesitancy scale: Psychometric properties and validation. Vaccine.

[23] Smith L.E., Amlôt R., Weinman J.A., Yiend J., Rubin G.J. (2017). A systematic review of factors affecting vaccine uptake in young children. Vaccine.

[24] Dror A.A., Eisenbach N., Taiber S., Morozov N.G., Mizrachi M., Zigron A., Srouji S., Sela E. (2020). Vaccine hesitancy: The next challenge in the fight against COVID-19. Eur. J. Epidemiol..

[25] Gobierno de Mexico Vacunación COVID Coronavirus. https://coronavirus.gob.mx/.

[26] Salud S. Cuestionario para la detección de riesgos a la salud mental COVID-19. https://www.misalud.unam.mx/covid19/.

[27] Delgado-Gallegos J.L., Padilla-Rivas G.R., Zúñiga-Violante E., Avilés-Rodríguez G., Arellanos-Soto D., Gastelum-Arias L.J., Villareal H.F., Cosío-León M.D.L., Romo-Cardenas G.S., Moreno-Treviño M.G. (2021). Determinants of COVID-19 Vaccine Hesitancy: A Cross-Sectional Study on a Mexican Population Using an Online Questionnaire (COV-AHQ). Front. Public Health.

[28] Taylor S., Landry C.A., Paluszek M.M., Fergus T.A., McKay D., Asmundson G.J.G. (2020). Development and initial validation of the COVID Stress Scales. J. Anxiety Disord..

[29] Delgado-Gallegos J., Montemayor-Garza R., Padilla-Rivas G., Franco-Villareal H., Islas J. (2020). Prevalence of Stress in Healthcare Professionals during the COVID-19 Pandemic in Northeast Mexico: A Remote, Fast Survey Evaluation, Using an Adapted COVID-19 Stress Scales. Int. J. Environ. Res. Public Health.

[30] Delgado-Gallegos J.L., Padilla-Rivas G.R., Zuñiga-Violante E., Avilés-Rodriguez G., Arellanos-Soto D., Villareal H.F., Cosío-León M.D.L., Romo-Cardenas G.S., Islas J.F. (2021). Teaching Anxiety, Stress and Resilience During the COVID-19 Pandemic: Evaluating the Vulnerability of Academic Professionals in Mexico Through the Adapted COVID-19 Stress Scales. Front. Public Health.

[31] Lwange S.K., Lemershow S. (1991). Sample Size Determination in Health Studies: A Practical Manual.

[32] Sharif A., Aloui C., Yarovaya L. (2020). COVID-19 pandemic, oil prices, stock market, geopolitical risk and policy uncertainty nexus in the US economy: Fresh evidence from the waveletbased approach. Int. Rev. Financ. Anal..

[33] Gobierno de Mexico Vacunación Contra COVID-19 Para Adolescentes de 12 A 17 Años. http://vacunacovid.gob.mx/wordpress/vacuna-covid19-adolescentes/.

[34] The Oxford Martin Programme. https://ourworldindata.org/covid-vaccinations?country=OWID_WRL.

[35] Kwok K.O., Li K.-K., Wei W.I., Tang A., Wong S.Y.S., Lee S.S. (2021). Influenza vaccine uptake, COVID-19 vaccination intention and vaccine hesitancy among nurses: A survey. Int. J. Nurs. Stud..

[36] Coustasse A., Kimble C., Maxik K. (2020). COVID-19 and Vaccine Hesitancy. J. Ambul. Care Manag..

[37] Forbes Detectan Aumento de 300% en la Demanda de Vacuna Contra Influenza. https://www.forbes.com.mx/noticias-aumento-300-demanda-vacuna-influenza/.

[38] He L., Zhou W., He M., Nie X., He J. (2021). Openness and COVID-19 induced xenophobia: The roles of trade and migration in sustainable development. PLoS ONE.

[39] Wang D., Gee G.C., Bahiru E., Yang E.H., Hsu J.J. (2020). Asian-Americans and Pacific Islanders in COVID-19: Emerging Disparities Amid Discrimination. J. Gen. Intern. Med..

[40] Farley J.H., Hines J., Lee N.K., Brooks S.E., Nair N., Brown C.L., Doll K.M., Sullivan E.J., Chapman-Davis E. (2020). Promoting health equity in the era of COVID-19. Gynecol. Oncol..

[41] Davies S. (2020). Pandemics and the consequences of COVID-19. Econ. Aff..

[42] Hilsenrath P.E. (2020). Ethics and Economic Growth in the Age of COVID-19: What Is a Just Society to Do?. J. Rural. Health.

[43] Ahorsu D.K., Lin C.-Y., Pakpour A.H. (2020). The Association Between Health Status and Insomnia, Mental Health, and Preventive Behaviors: The Mediating Role of Fear of COVID-19. Gerontol. Geriatr. Med..

[44] Han M.F.Y., Mahendran R., Yu J. (2021). Associations Between Fear of COVID-19, Affective Symptoms and Risk Perception Among Community-Dwelling Older Adults During a COVID-19 Lockdown. Front. Psychol..

[45] Mistry S.K., Ali A.R.M.M., Akther F., Yadav U.N., Harris M.F. (2021). Exploring fear of COVID-19 and its correlates among older adults in Bangladesh. Glob. Health.

[46] Caycho-Rodríguez T., Tomás J.M., Barboza-Palomino M., Ventura-León J., Gallegos M., Reyes-Bossio M., Vilca L.W. (2021). Assessment of Fear of COVID-19 in Older Adults: Validation of the Fear of COVID-19 Scale. Int. J. Ment. Health Addict..

[47] Kamidani S., Rostad C.A., Anderson E.J. (2020). COVID-19 vaccine development: A pediatric perspective. Curr. Opin. Pediatr..

[48] Balasubramanian S., Rao N.M., Goenka A., Roderick M., Ramanan A.V. (2020). Coronavirus Disease 2019 (COVID-19) in Children-What We Know So Far and What We Do Not. Indian Pediatr..

[49] Doubova S.V., García-Saisó S., Perez-Cuevas R., Sarabia-González O., Pacheco-Estrello P., Leslie H.H., Santamaría C., Torres-Arreola L.D.P., Infante-Castañeda C. (2018). Barriers and opportunities to improve the foundations for high-quality healthcare in the Mexican Health System. Health Policy Plan..

[50] Khubchandani J., Sharma S., Price J.H., Wiblishauser M.J., Sharma M., Webb F.J. (2021). COVID-19 Vaccination Hesitancy in the United States: A Rapid National Assessment. J. Community Health.

[51] Baptista Lucio P., Almazán Zimerman A., Alberto Loeza Altamirano C., Cloezaa M., Víctor Alfonso López Alcaraz U., Luis Cárdenas Domínguez J. (2020). Encuesta nacional a docentes ante el COVID-19. Retos para la educación a distancia National Survey to Teachers Facing COVID-19. Challenges for Distance Education. Rev. Latinoam. Estud. Educ..

[52] Allen J.D., Feng W., Corlin L., Porteny T., Acevedo A., Schildkraut D., King E., Ladin K., Fu Q., Stopka T.J. (2021). Why are some people reluctant to be vaccinated for COVID-19? A cross-sectional survey among U.S. Adults in May-June 2020. Prev. Med. Rep..

